# Crystal structure of bis­(benzoato-κ*O*)di­butyl­tin(IV), nBu_2_Sn(bzo)_2_

**DOI:** 10.1107/S2056989016008604

**Published:** 2016-06-03

**Authors:** Hans Reuter, Coco K. Y. A. Okio

**Affiliations:** a Institute of Chemistry of New Materials, University of Osnabrück, Barbarastrasse 7, 49069 Osnabrück, Germany; b Departamento de Química, Facultad de Ciencias, Universidad Nacional de Colombia, Carerra 30 No 45-03, Bogotá, Colombia

**Keywords:** crystal structure, diorganotin(IV) dibenzoate, unsymmetrical bidentate bonding

## Abstract

The title compound, [Sn(C_4_H_9_)_2_(C_6_H_5_COO)_2_], was synthesized in order to study the inter­action between di-*n*-butyl­tin(IV) oxide and some carb­oxy­lic acids. Di-*n*-butyl­tin(IV) dibenzoate, nBu_2_Sn(obz)_2_, exhibits the same structural features as other diorganotin(IV) dibenzoates characterized by an unsymmetrical bidentate bonding mode [Δ(Sn—O) ≃ 0.4 Å] of the two benzoate groups to tin. In a first approximation, the coordination sphere at tin resulting from the two stronger bonded O atoms [2.1227 (17) and 2.1405 (16) Å] and the two α-C atoms of the *n*-butyl groups [2.125 (3) and 2.129 (2) Å] is compressed to a tetra­gonal disphenoid [〈(C—Sn—C) = 148.2 (1)° and 〈(O—Sn—O) = 82.01 (6)°]. This coordination sphere is expanded by the less strongly bonded two O atoms [2.507 (2) and 2.485 (2) Å] to a substanti­ally distorted octa­hedron and by a weak inter­molecular Sn⋯O inter­action [2.943 (2) Å] to a penta­gonal bipyramid with the formation of centrosymmetric dimers. The unbranched butyl groups adopt two different conformations: *anti–gauche* [torsion angles: 166.0 (2)–63.9 (4)°] and *gauche–gauche* [65.0 (3)–56.3 (3)°]. Inter­molecular inter­actions between the dimers are restricted to O⋯H—C contacts (2.64 Å) and van der Waals inter­actions.

## Abstract 2

The title compound, [Sn(C_4_H_9_)_2_(C_6_H_5_COO)_2_], was synthesized in order to study the inter­action between di-*n*-butyl­tin(IV) oxide and some carb­oxy­lic acids. Di-*n*-butyl­tin(IV) dibenzoate, nBu_2_Sn(obz)_2_, exhibits the same structural features as other diorganotin(IV) dibenzoates characterized by an unsymmetrical bidentate bonding mode [Δ(Sn—O) ≃ 0.4 Å] of the two benzoate groups to tin.

## Chemical context   

Organotin(IV) complexes have been studied extensively because of the diversity of structures that such compounds can form and their potential biological activities as well as their wide industrial and agricultural applications (Davies & Smith, 1982 ▸). As part of our inter­est in this type of complex (Cortés *et al.*, 2011 ▸), we describe here the synthesis of the di-*n*-butyl­tin(IV) title complex with benzoic anions as ligands. The structures of some di-*n*-butyl­tin carboxyl­ates have been reported previously by Kemmer *et al.* (2000 ▸) and Win *et al.* (2015 ▸).
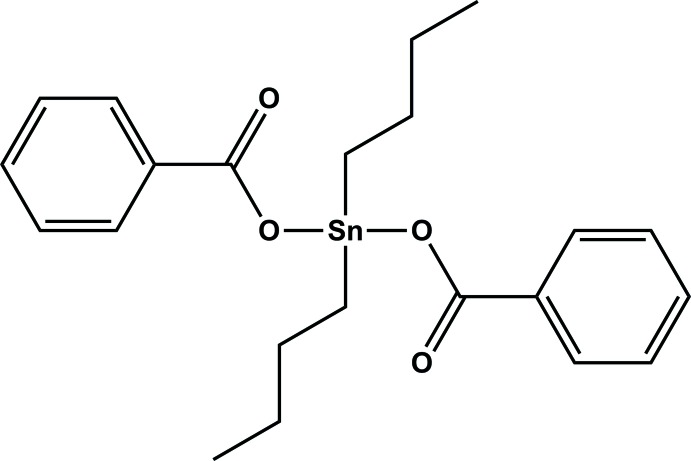


## Structural commentary   

**Figure 1:**
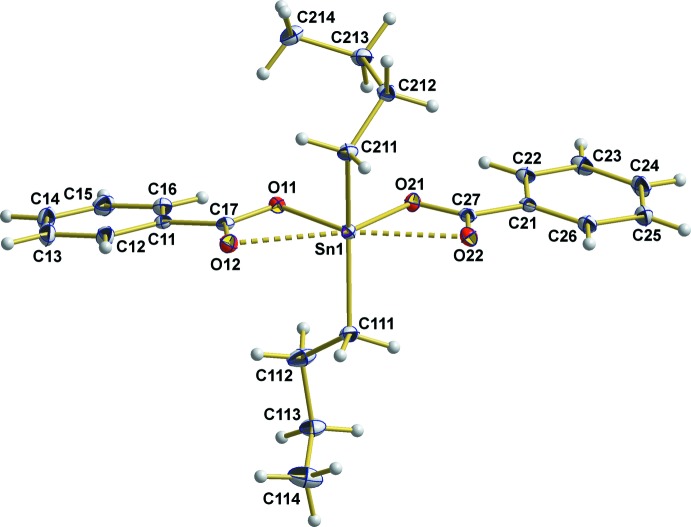
The asymmetric unit of the title compound, showing the atom-labeling scheme and displacement ellipsoids for the non-H atoms at the 50% probability level.

The asymmetric unit of the title compound (Fig. 1 ▸) consists of one mol­ecule of the title compound with all atoms in general positions. The two Sn—C bonds are of equal length within the limits of standard deviations [2.125 (3), 2.129 (2) Å, mean value: 2.127 (2) Å]. Within the *n*-butyl groups, the C—C bond lengths are in the range 1.480 (4)–1.527 (4) Å [mean value 1.52 (2) Å] in the first group [C111–C114] and 1.523 (3)–1.528 (3) Å [mean value: 1.523 (3) Å] in the second [C211–C214]. While the C—C bonds of the latter *n*-butyl group correspond very well with the value [1.524 (14) Å] calculated for C*sp* ^3^—C*sp* ^3^ bonds by Allen *et al.* (1987 ▸), the somewhat shorter C—C bonds of the first *n*-butyl group are strongly influenced by some larger anisotropic displacement ellipsoids of the carbon atoms as a result of thermal motion or unresolved static disorder. Bond angles at the carbon atoms of the *n*-butyl groups range from 114.1 (2)–115.0 (3)°, and 114.0 (2)–116.0 (2)°, respectively. The two *n*-butyl groups, however, adopt different conformations: *anti–gauche* [166.0 (2)–63.9 (4)°] for the first one [*n* = 1] and *gauche–gauche* [65.0 (3)–56.3 (3)°] for the second one [*n* = 2], both with respect to the C*n*11—C*n*12 and C*n*12—C*n*13 bonds.

**Figure 2:**
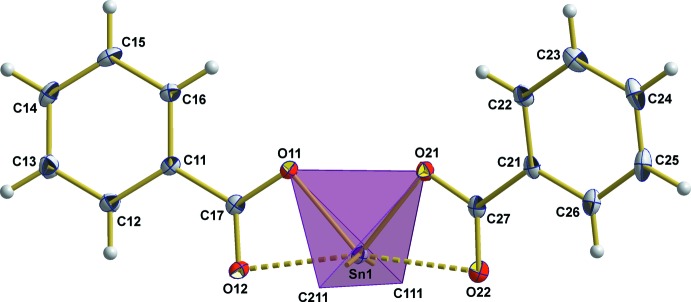
Polyhedron model of the coordination sphere of the tin atom; *n*-butyl groups have been omitted for clarity, weak Sn⋯O inter­actions are indicated by dashed lines. Displacement ellipsoids are shown at the 50% probability level.

The two carboxyl­ate ligands coordinate to the Sn^IV^ atom asymmetrically. One oxygen atom of each carboxyl­ate group reveals a very strong/short Sn—O bond of 2.122 (17) and 2.1405 (16) Å, respectively, which are of similar strength as the Sn—C bonds. With respect to these four strong bonds, the coordination polyhedron at the tin atom is compressed to a tetra­gonal disphenoid (Fig. 2 ▸) with a bond angle of 148.2 (1)° between the two α-carbon atoms of the *n*-butyl groups and of 82.01 (6)° between the two oxygen atoms of the benzoate groups. On the carboxyl­ate side, the corresponding C—O bonds are long [1.299 (3)/1.287 (3) Å] in accordance with localized single bonds. The second oxygen atom of each carboxyl­ate group exhibits a much weaker coordination to the tin atom [2.485 (2)/2.507 (2) Å], giving rise to a strongly distorted octa­hedral coordination with the *n*-butyl groups in the *trans*-position. Besides, its corresponding C—O bonds are significantly shorter [1.238 (3)/1.244 (3) Å], indicating a C=O double bond.

The phenyl groups are almost planar with mean C—C bond lengths of 1.387 (5) Å and bond angles of 120.0 (5)°. Again, the bond lengths are in good agreement with the literature data (Allen *et al.*, 1987 ▸) of 1.387 (10) Å for C_ar_—C_ar_. The phenyl rings subtend dihedral angles of 6.7 (2) and 6.4 (3)° with the planes formed by the three atoms of the carboxyl­ate groups, while the dihedral angle between the phenyl rings is 17.7 (1)°. As usual, the C—C single bonds between the carboxyl­ate and phenyl groups are somewhat shorter [1.489 (3), 1.487 (3) Å] than the C—C single bonds between *sp* ^3^-hybridized carbon atoms (see above).

## Supra­molecular features   

**Figure 3:**
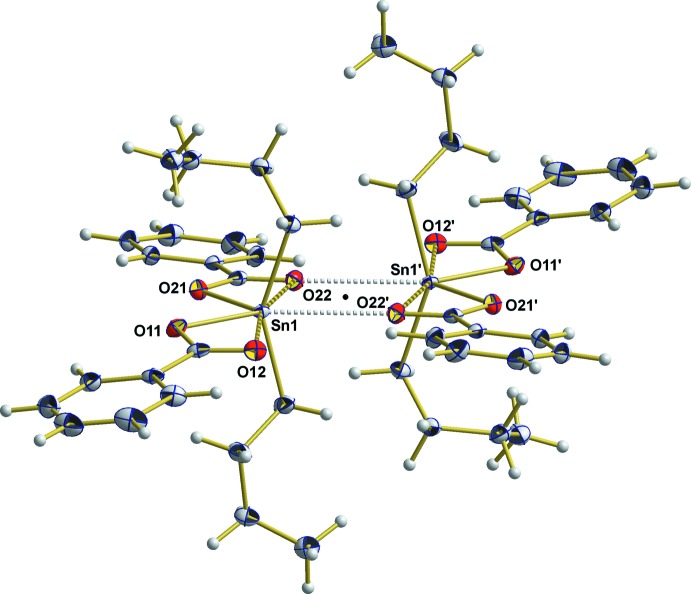
Centrosymmetric (center of symmetry = black dot) dimers of the title compound resulting from weak inter­molecular Sn⋯O inter­actions (grey dashed lines). [Symmetry code: (′) 2 − *x*, 1 − *y*, 1 − *z*.]

**Figure 4:**
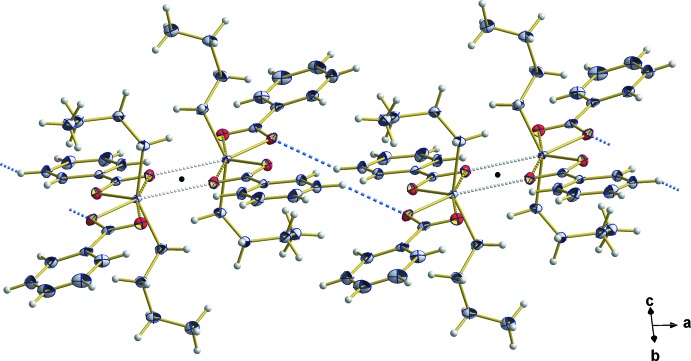
C—H⋯O inter­actions (blue dashed lines) between neighboring dimers responsible for their chain-shaped arrangement along the *a* axis.

**Figure 5:**
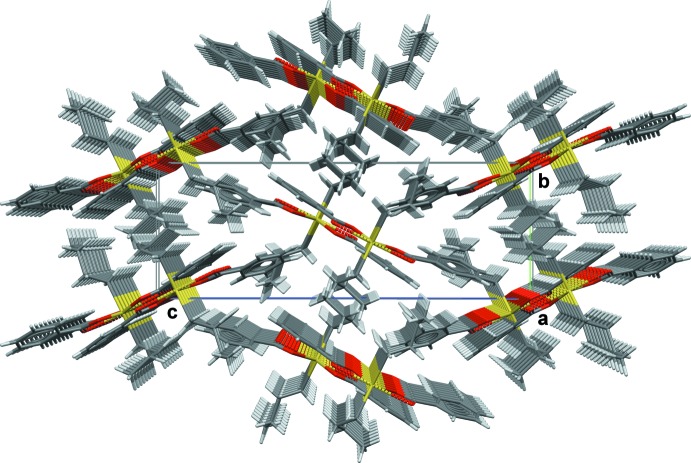
Perspective view of the crystal structure of the title compound viewed down the *a* axis.

Besides the described intra­molecular Sn—O inter­actions responsible for the distorted octa­hedral coordination of the tin atom, some weak inter­molecular Sn⋯O inter­actions of 2.943 (2) Å exist and lead to the formation of centrosymmetric dimers and hence the coordination sphere of the tin atom is expanded from six, octa­hedral to seven, penta­gonal–biypramidal (Fig. 3 ▸). Once the coordination sphere of the tin atom is completed, the solid-state packing of these dimers is due exclusively to inter­molecular O⋯H—C contacts [O11⋯H23^i^ = 2.64 Å; symmetry code: (i) 1 − *x*, 1 − *y*, 1 − *z*] and van der Waals inter­actions (Fig. 4 ▸), respectively, while π–π stacking can be excluded (Fig. 5 ▸).

## Database survey   

Structures of diorganotin(IV) di­carboxyl­ates, *R*_2_Sn(O_2_C*R*′)_2_, have been intensively studied, including di-*n*-butyl ones (*R* = *n*-Bu) (*i.e.* Kemmer *et al.*, 2000 ▸; Win *et al.*, 2015 ▸), but up to now only the structures of two dibenzoates (*R*′ = Ph), with *R* = Me (Tiekink, 1991 ▸) and *R* = Et,Ph (Amini *et al.*, 2009 ▸), were known. Both exhibit the same structural features as the title compound but some differences arise with respect to bond lengths and angles (Et,Ph/Me): *d*(Sn—C) = 2.128 (3), 2.124 (4) Å/2.10 (2), 2.10 (2) Å]_;_ *d*(Sn—O)_strong_: 2.150 (2), 2.153 (2)/2.156 (9), 2.128 (8) Å; 〈(C—Sn—C): 154.9 (1)/147.2 (7)°, 〈(O—Sn—O): 84.44 (7)/84.4 (4)°; *d*(Sn—O)_weak_: 2.400 (2), 2.551 (2)/2.51 (1), 2.510 (9) Å; *d*(Sn⋯O)_inter­molecular_: 2.812 (2)/2.955 (10) Å.

## Synthesis and crystallization   

The title compound was obtained by reacting 0.300 g (1.2 mmol) of di-*n*-butyl­tin oxide with 0.94 g (2.4 mmol) of benzoic acid in ethanol under reflux for 3.5 h. Colourless crystals suitable for X-ray analysis were grown by slow solvent evaporation. Elemental analysis calculated/found (%): C 55.61/55.38, H 5.94/5.66, ^1^H NMR (250 MHz, CDCl_3_), δ (p.p.m.): 8.19 (2H_*ortho*_, *D*, 7.25 Hz), 7.62 (1H_*para*_, *T*, 7.3 Hz), 7.50 (2H_*meta*_, *T*, 7.3 Hz), 1.89–1.72 (2H_α_ + 2H_β_, multiplets not resolved), 1.45 (2Hγ, *Hex*, 7.25 Hz), 0.92 (3H_δ_, *T*, 7.25 Hz); {^1^H}-^13^C NMR (250 MHz, CDCl_3_), δ (p.p.m.), *^n^J*(^13^C–^119/117^Sn) (Hz): 176.07 (–COO^−^), 133.10 (C_*para*_), 130.50 (C_*ortho*_), 130.12 C(_*ipso*_) 128.28 (C_*meta*_), 26.68 (C_β_) 35.3 (^2^ *J*), 26.32 (Cγ) 100.0/95.4 (^3^ *J*), 25.47 (C_α_) 584.2/558.6 (^1^ *J*), 13.48 (C_δ_), IR (ATR) ν (cm^−1^): 2965 (*m*), 2929 (*m*), 2865 (*w*), 1599 (*s*), 1556 (*s*), 1493 (*m*), 1450 (*m*), 1368 (*vs*, *br*), 1302 (*m*), 1251 (*m*), 1174 (*m*), 1134 (*m*), 1070 (*m*), 1024 (*m*), 861 (*s*), 717 (*vs*), 683 (*vs*), 541 (m), 449 (*s*), Raman ν (cm^−1^): 3071 (*m*), 2976 (*w*), 2935 (*m*), 2875 (*m*), 2859 (*m*), 1603 (*s*), 1567 (*w*), 1495 (*w*), 1451 (*m*), 1389 (*m*, *br*), 1180 (*w*), 1161 (*m*), 1137 (*w*), 1027 (*w*), 1003 (*s*), 869 (*m*), 616 (*m*), 519 (*m*), 409 (*w*), 282 (*w*), 216 (*m*), 161 (*w*), 84 (*vs*).

## Refinement   

Crystal data, data collection and structure refinement details are summarized in Table 1 ▸. Hydrogen atoms were clearly identified in difference Fourier syntheses. Their positions were calculated assuming idealized geometries and allowed to ride on the carbon atoms with C*–*-H = 0.98 Å (–CH_3_), 0.99 Å (–CH_2_–), and 0.95 Å (C—H_arom_) using one common isotropic displacement parameter for each *n*-butyl and phenyl group.

## Supplementary Material

Crystal structure: contains datablock(s) I. DOI: 10.1107/S2056989016008604/zl2663sup1.cif

Structure factors: contains datablock(s) I. DOI: 10.1107/S2056989016008604/zl2663Isup2.hkl

CCDC reference: 1482358

Additional supporting information: crystallographic information; 3D view; checkCIF report

## Figures and Tables

**Table 1:** Experimental details

Crystal data	
Chemical formula	[Sn(C_4_H_9_)_2_(C_7_H_5_O_2_)_2_]
*M*_r_	475.13
Crystal system, space group	Monoclinic, *P*2_1_/*c*
Temperature (K)	100
*a*, *b*, *c* (Å)	11.6801 (5), 8.1098 (3), 22.6345 (8)
β (°)	98.736 (2)
*V* (Å^3^)	2119.14 (14)
*Z*	4
Radiation type	Mo *K*α
μ (mm^−1^)	1.23
Crystal size (mm)	0.38 × 0.37 × 0.21

Data collection	
Diffractometer	Bruker APEXII CCD
Absorption correction	Multi-scan (*SADABS*; Bruker, 2009 ▸)
*T*_min_, *T*_max_	0.654, 0.782
No. of measured, independent and observed [*I* > 2σ(*I*)] reflections	148965, 5116, 4751
*R*_int_	0.058
(sin θ/λ)_max_ (Å^−1^)	0.661

Refinement	
*R*[*F* ^2^ > 2σ(*F* ^2^)], *wR*(*F* ^2^), *S*	0.028, 0.070, 1.20
No. of reflections	5116
No. of parameters	250
H-atom treatment	H-atom parameters constrained
Δρ_max_, Δρ_min_ (e Å^−3^)	1.25, −0.83

Computer programs: *APEX2* and *SAINT* (Bruker, 2009 ▸), *SHELXS97* and *SHELXTL* (Sheldrick, 2008 ▸), *SHELXL2014* (Sheldrick, 2015 ▸), *DIAMOND* (Brandenburg, 2006 ▸) and *Mercury* (Macrae *et al.*, 2008 ▸).

## supplementary crystallographic information

### Crystal data

**Table:**

[Sn(C_4_H_9_)_2_(C_7_H_5_O_2_)_2_]	*F*(000) = 968
*M**_r_* = 475.13	*D*_x_ = 1.489 Mg m^−^^3^
Monoclinic, *P*2_1_/*c*	Mo *K*α radiation, λ = 0.71073 Å
*a* = 11.6801 (5) Å	Cell parameters from 8832 reflections
*b* = 8.1098 (3) Å	θ = 2.3–28.1°
*c* = 22.6345 (8) Å	µ = 1.23 mm^−^^1^
β = 98.736 (2)°	*T* = 100 K
*V* = 2119.14 (14) Å^3^	Bloc, colourless
*Z* = 4	0.38 × 0.37 × 0.21 mm

### Data collection

**Table:**

Bruker APEXII CCD diffractometer	4751 reflections with *I* > 2σ(*I*)
φ and ω scans	*R*_int_ = 0.058
Absorption correction: multi-scan (SADABS; Bruker, 2009)	θ_max_ = 28.0°, θ_min_ = 2.3°
*T*_min_ = 0.654, *T*_max_ = 0.782	*h* = −15→15
148965 measured reflections	*k* = −10→10
5116 independent reflections	*l* = −29→29

### Refinement

**Table:**

Refinement on *F*^2^	0 restraints
Least-squares matrix: full	Hydrogen site location: inferred from neighbouring sites
*R*[*F*^2^ > 2σ(*F*^2^)] = 0.028	H-atom parameters constrained
*wR*(*F*^2^) = 0.070	*w* = 1/[σ^2^(*F*_o_^2^) + (0.0242*P*)^2^ + 3.7196*P*] where *P* = (*F*_o_^2^ + 2*F*_c_^2^)/3
*S* = 1.20	(Δ/σ)_max_ = 0.003
5116 reflections	Δρ_max_ = 1.25 e Å^−^^3^
250 parameters	Δρ_min_ = −0.83 e Å^−^^3^

### Special details

**Table:**

Geometry. All esds (except the esd in the dihedral angle between two l.s. planes) are estimated using the full covariance matrix. The cell esds are taken into account individually in the estimation of esds in distances, angles and torsion angles; correlations between esds in cell parameters are only used when they are defined by crystal symmetry. An approximate (isotropic) treatment of cell esds is used for estimating esds involving l.s. planes.

### Fractional atomic coordinates and isotropic or equivalent isotropic displacement parameters (Å^2^)

**Table:**

	*x*	*y*	*z*	*U*_iso_*/*U*_eq_	
Sn1	0.85118 (2)	0.41711 (2)	0.42877 (2)	0.01431 (6)	
C111	0.9080 (2)	0.6348 (3)	0.38974 (11)	0.0221 (5)	
H111	0.9084	0.7266	0.4186	0.055 (4)*	
H112	0.9886	0.6182	0.3824	0.055 (4)*	
C112	0.8348 (3)	0.6837 (4)	0.33200 (14)	0.0329 (7)	
H113	0.7521	0.6733	0.3366	0.055 (4)*	
H114	0.8497	0.6058	0.3004	0.055 (4)*	
C113	0.8566 (2)	0.8591 (3)	0.31174 (13)	0.0279 (6)	
H115	0.7996	0.8844	0.2759	0.055 (4)*	
H116	0.8424	0.9365	0.3437	0.055 (4)*	
C114	0.9743 (3)	0.8894 (4)	0.29746 (16)	0.0412 (8)	
H117	0.9880	0.8177	0.2644	0.055 (4)*	
H118	1.0317	0.8656	0.3327	0.055 (4)*	
H119	0.9811	1.0050	0.2859	0.055 (4)*	
C211	0.8942 (2)	0.1842 (3)	0.46968 (11)	0.0194 (5)	
H211	0.8823	0.0983	0.4383	0.029 (3)*	
H212	0.9777	0.1849	0.4863	0.029 (3)*	
C212	0.8263 (2)	0.1349 (3)	0.51951 (11)	0.0215 (5)	
H213	0.8367	0.2219	0.5505	0.029 (3)*	
H214	0.8602	0.0321	0.5381	0.029 (3)*	
C213	0.6965 (2)	0.1076 (3)	0.50023 (12)	0.0236 (5)	
H215	0.6618	0.0722	0.5354	0.029 (3)*	
H216	0.6604	0.2138	0.4862	0.029 (3)*	
C214	0.6678 (2)	−0.0201 (3)	0.45088 (13)	0.0265 (6)	
H217	0.7129	−0.1205	0.4616	0.029 (3)*	
H218	0.6872	0.0241	0.4133	0.029 (3)*	
H219	0.5850	−0.0461	0.4459	0.029 (3)*	
C11	0.6734 (2)	0.2748 (3)	0.26476 (10)	0.0177 (5)	
C12	0.7197 (2)	0.2006 (3)	0.21811 (11)	0.0237 (5)	
H12	0.7993	0.1711	0.2230	0.036 (4)*	
C13	0.6481 (3)	0.1703 (4)	0.16446 (12)	0.0308 (6)	
H13	0.6789	0.1191	0.1325	0.036 (4)*	
C14	0.5326 (3)	0.2137 (4)	0.15702 (12)	0.0310 (6)	
H14	0.4841	0.1906	0.1203	0.036 (4)*	
C15	0.4874 (2)	0.2905 (4)	0.20272 (12)	0.0280 (6)	
H15	0.4083	0.3227	0.1971	0.036 (4)*	
C16	0.5574 (2)	0.3210 (3)	0.25696 (11)	0.0216 (5)	
H16	0.5262	0.3732	0.2886	0.036 (4)*	
C17	0.7499 (2)	0.3071 (3)	0.32243 (10)	0.0176 (5)	
O11	0.70125 (15)	0.3642 (2)	0.36615 (7)	0.0180 (3)	
O12	0.85583 (15)	0.2840 (2)	0.32918 (8)	0.0208 (4)	
C21	0.7408 (2)	0.6684 (3)	0.56956 (10)	0.0149 (4)	
C22	0.6211 (2)	0.6786 (3)	0.56612 (11)	0.0181 (5)	
H22	0.5720	0.6277	0.5339	0.025 (4)*	
C23	0.5736 (2)	0.7631 (3)	0.60976 (11)	0.0222 (5)	
H23	0.4920	0.7678	0.6083	0.025 (4)*	
C24	0.6458 (2)	0.8408 (3)	0.65557 (12)	0.0251 (5)	
H24	0.6132	0.9007	0.6850	0.025 (4)*	
C25	0.7647 (2)	0.8325 (4)	0.65901 (12)	0.0251 (5)	
H25	0.8134	0.8867	0.6905	0.025 (4)*	
C26	0.8129 (2)	0.7444 (3)	0.61626 (11)	0.0203 (5)	
H26	0.8947	0.7361	0.6189	0.025 (4)*	
C27	0.7927 (2)	0.5767 (3)	0.52329 (10)	0.0156 (4)	
O21	0.72511 (14)	0.5203 (2)	0.47756 (7)	0.0170 (3)	
O22	0.89909 (15)	0.5548 (2)	0.52750 (8)	0.0187 (3)	

### Atomic displacement parameters (Å^2^)

**Table:**

	*U*^11^	*U*^22^	*U*^33^	*U*^12^	*U*^13^	*U*^23^
Sn1	0.01604 (8)	0.01393 (8)	0.01351 (8)	−0.00007 (6)	0.00406 (6)	0.00020 (6)
C111	0.0236 (12)	0.0225 (12)	0.0194 (12)	−0.0090 (10)	0.0008 (9)	0.0034 (10)
C112	0.0312 (15)	0.0229 (14)	0.0400 (16)	−0.0061 (12)	−0.0097 (12)	0.0138 (12)
C113	0.0294 (14)	0.0190 (12)	0.0350 (15)	0.0017 (11)	0.0042 (11)	0.0082 (11)
C114	0.0306 (15)	0.0429 (19)	0.051 (2)	0.0022 (14)	0.0093 (14)	0.0224 (16)
C211	0.0193 (11)	0.0160 (11)	0.0227 (12)	0.0016 (9)	0.0026 (9)	0.0022 (9)
C212	0.0245 (12)	0.0203 (12)	0.0188 (11)	−0.0028 (10)	0.0007 (9)	0.0040 (9)
C213	0.0242 (12)	0.0211 (13)	0.0267 (13)	−0.0036 (10)	0.0076 (10)	0.0031 (10)
C214	0.0265 (13)	0.0182 (12)	0.0331 (14)	−0.0034 (10)	−0.0008 (11)	0.0023 (11)
C11	0.0245 (12)	0.0121 (11)	0.0163 (11)	−0.0047 (9)	0.0027 (9)	0.0012 (8)
C12	0.0327 (14)	0.0172 (12)	0.0214 (12)	0.0015 (10)	0.0050 (10)	−0.0024 (10)
C13	0.0514 (18)	0.0213 (13)	0.0194 (12)	0.0006 (13)	0.0047 (12)	−0.0061 (10)
C14	0.0442 (17)	0.0250 (14)	0.0203 (12)	−0.0076 (12)	−0.0058 (11)	−0.0018 (11)
C15	0.0271 (13)	0.0277 (14)	0.0272 (14)	−0.0054 (11)	−0.0020 (11)	0.0028 (11)
C16	0.0228 (12)	0.0224 (12)	0.0201 (12)	−0.0049 (10)	0.0042 (9)	0.0001 (10)
C17	0.0241 (12)	0.0118 (10)	0.0176 (11)	−0.0033 (9)	0.0054 (9)	0.0018 (8)
O11	0.0199 (8)	0.0189 (8)	0.0160 (8)	−0.0032 (7)	0.0055 (6)	−0.0021 (7)
O12	0.0234 (9)	0.0192 (9)	0.0202 (8)	0.0006 (7)	0.0045 (7)	−0.0003 (7)
C21	0.0173 (10)	0.0132 (10)	0.0155 (10)	0.0007 (8)	0.0063 (8)	0.0034 (8)
C22	0.0167 (11)	0.0184 (11)	0.0194 (11)	0.0006 (9)	0.0037 (9)	0.0019 (9)
C23	0.0189 (11)	0.0242 (13)	0.0255 (12)	0.0061 (10)	0.0102 (9)	0.0046 (10)
C24	0.0314 (14)	0.0237 (13)	0.0233 (12)	0.0042 (11)	0.0143 (11)	−0.0012 (10)
C25	0.0278 (13)	0.0279 (14)	0.0208 (12)	−0.0053 (11)	0.0077 (10)	−0.0052 (10)
C26	0.0191 (11)	0.0235 (12)	0.0192 (11)	−0.0020 (10)	0.0057 (9)	−0.0004 (10)
C27	0.0193 (11)	0.0126 (10)	0.0156 (10)	0.0002 (9)	0.0051 (8)	0.0037 (8)
O21	0.0170 (8)	0.0165 (8)	0.0179 (8)	0.0019 (7)	0.0036 (6)	−0.0019 (6)
O22	0.0172 (8)	0.0210 (9)	0.0187 (8)	0.0030 (7)	0.0052 (6)	−0.0002 (7)

### Geometric parameters (Å, º)

**Table:**

Sn1—O11	2.1227 (17)	C214—H219	0.9800
Sn1—C111	2.125 (3)	C11—C16	1.390 (4)
Sn1—C211	2.129 (2)	C11—C12	1.394 (3)
Sn1—O21	2.1405 (16)	C11—C17	1.489 (3)
Sn1—O22	2.4846 (17)	C12—C13	1.388 (4)
Sn1—O12	2.5071 (17)	C12—H12	0.9500
Sn1—C17	2.669 (2)	C13—C14	1.379 (4)
C111—C112	1.502 (4)	C13—H13	0.9500
C111—H111	0.9900	C14—C15	1.380 (4)
C111—H112	0.9900	C14—H14	0.9500
C112—C113	1.527 (4)	C15—C16	1.390 (4)
C112—H113	0.9900	C15—H15	0.9500
C112—H114	0.9900	C16—H16	0.9500
C113—C114	1.480 (4)	C17—O12	1.238 (3)
C113—H115	0.9900	C17—O11	1.299 (3)
C113—H116	0.9900	C21—C26	1.391 (3)
C114—H117	0.9800	C21—C22	1.392 (3)
C114—H118	0.9800	C21—C27	1.487 (3)
C114—H119	0.9800	C22—C23	1.385 (3)
C211—C212	1.528 (3)	C22—H22	0.9500
C211—H211	0.9900	C23—C24	1.385 (4)
C211—H212	0.9900	C23—H23	0.9500
C212—C213	1.528 (4)	C24—C25	1.381 (4)
C212—H213	0.9900	C24—H24	0.9500
C212—H214	0.9900	C25—C26	1.389 (3)
C213—C214	1.523 (4)	C25—H25	0.9500
C213—H215	0.9900	C26—H26	0.9500
C213—H216	0.9900	C27—O22	1.244 (3)
C214—H217	0.9800	C27—O21	1.287 (3)
C214—H218	0.9800		
			
O11—Sn1—C111	99.70 (8)	C212—C213—H215	108.8
O11—Sn1—C211	103.14 (8)	C214—C213—H216	108.8
C111—Sn1—C211	148.17 (10)	C212—C213—H216	108.8
O11—Sn1—O21	82.01 (6)	H215—C213—H216	107.7
C111—Sn1—O21	99.92 (9)	C213—C214—H217	109.5
C211—Sn1—O21	104.93 (8)	C213—C214—H218	109.5
O11—Sn1—O22	137.87 (6)	H217—C214—H218	109.5
C111—Sn1—O22	87.63 (8)	C213—C214—H219	109.5
C211—Sn1—O22	90.07 (8)	H217—C214—H219	109.5
O21—Sn1—O22	55.87 (6)	H218—C214—H219	109.5
O11—Sn1—O12	56.01 (6)	C16—C11—C12	120.2 (2)
C111—Sn1—O12	86.17 (8)	C16—C11—C17	120.5 (2)
C211—Sn1—O12	88.47 (8)	C12—C11—C17	119.3 (2)
O21—Sn1—O12	137.95 (6)	C13—C12—C11	119.1 (3)
O22—Sn1—O12	165.78 (6)	C13—C12—H12	120.4
O11—Sn1—C17	28.66 (7)	C11—C12—H12	120.4
C111—Sn1—C17	91.51 (8)	C14—C13—C12	120.7 (3)
C211—Sn1—C17	97.98 (9)	C14—C13—H13	119.7
O21—Sn1—C17	110.51 (7)	C12—C13—H13	119.7
O22—Sn1—C17	165.85 (7)	C13—C14—C15	120.2 (3)
O12—Sn1—C17	27.45 (7)	C13—C14—H14	119.9
C112—C111—Sn1	114.12 (17)	C15—C14—H14	119.9
C112—C111—H111	108.7	C14—C15—C16	120.1 (3)
Sn1—C111—H111	108.7	C14—C15—H15	119.9
C112—C111—H112	108.7	C16—C15—H15	119.9
Sn1—C111—H112	108.7	C15—C16—C11	119.7 (2)
H111—C111—H112	107.6	C15—C16—H16	120.2
C111—C112—C113	114.1 (2)	C11—C16—H16	120.2
C111—C112—H113	108.7	O12—C17—O11	120.3 (2)
C113—C112—H113	108.7	O12—C17—C11	122.5 (2)
C111—C112—H114	108.7	O11—C17—C11	117.2 (2)
C113—C112—H114	108.7	O12—C17—Sn1	68.98 (13)
H113—C112—H114	107.6	O11—C17—Sn1	51.62 (11)
C114—C113—C112	115.0 (3)	C11—C17—Sn1	166.98 (18)
C114—C113—H115	108.5	C17—O11—Sn1	99.72 (14)
C112—C113—H115	108.5	C17—O12—Sn1	83.58 (14)
C114—C113—H116	108.5	C26—C21—C22	120.2 (2)
C112—C113—H116	108.5	C26—C21—C27	119.5 (2)
H115—C113—H116	107.5	C22—C21—C27	120.3 (2)
C113—C114—H117	109.5	C23—C22—C21	119.8 (2)
C113—C114—H118	109.5	C23—C22—H22	120.1
H117—C114—H118	109.5	C21—C22—H22	120.1
C113—C114—H119	109.5	C24—C23—C22	119.7 (2)
H117—C114—H119	109.5	C24—C23—H23	120.2
H118—C114—H119	109.5	C22—C23—H23	120.2
C212—C211—Sn1	116.01 (17)	C25—C24—C23	120.9 (2)
C212—C211—H211	108.3	C25—C24—H24	119.6
Sn1—C211—H211	108.3	C23—C24—H24	119.6
C212—C211—H212	108.3	C24—C25—C26	119.8 (2)
Sn1—C211—H212	108.3	C24—C25—H25	120.1
H211—C211—H212	107.4	C26—C25—H25	120.1
C211—C212—C213	115.5 (2)	C25—C26—C21	119.6 (2)
C211—C212—H213	108.4	C25—C26—H26	120.2
C213—C212—H213	108.4	C21—C26—H26	120.2
C211—C212—H214	108.4	O22—C27—O21	119.6 (2)
C213—C212—H214	108.4	O22—C27—C21	121.8 (2)
H213—C212—H214	107.5	O21—C27—C21	118.6 (2)
C214—C213—C212	114.0 (2)	C27—O21—Sn1	99.65 (14)
C214—C213—H215	108.8	C27—O22—Sn1	84.80 (14)
			
C111—C112—C113—C114	63.9 (4)	Sn1—C111—C112—C113	166.0 (2)
C211—C212—C213—C214	56.3 (3)	Sn1—C211—C212—C213	65.0 (3)
